# Garnering Partnerships to Bridge Gaps Among Mental Health, Health Care, and Public Health

**Published:** 2009-12-15

**Authors:** Letitia Presley-Cantrell, Elsie Freeman, Valerie J. Edwards, Sharrice White-Cooper, Stephanie Sturgis, Janet B. Croft, Kenneth S. Thompson

**Affiliations:** Centers for Disease Control and Prevention; Maine Department of Health and Human Services, Augusta, Maine; Centers for Disease Control and Prevention, Atlanta, Georgia; Centers for Disease Control and Prevention, Atlanta, Georgia; Centers for Disease Control and Prevention, Atlanta, Georgia; Centers for Disease Control and Prevention, Atlanta, Georgia; Substance Abuse and Mental Health Services Administration, Rockville, Maryland

## Abstract

Integrating mental health and public health chronic disease programs requires partnerships at all government levels. Four examples illustrate this approach: 1) a federal partnership to implement mental health and mental illness modules in the Behavioral Risk Factor Surveillance System; 2) a state partnership to improve diabetes health outcomes for people with mental illness; 3) a community-level example of a partnership with local aging and disability agencies to modify a home health service to reduce depression and improve quality of life among isolated, chronically ill seniors; and 4) a second community-level example of a partnership to promote depression screening and management and secure coverage in primary care settings. Integration of mental health and chronic disease public health programs is a challenging but essential and achievable task in protecting Americans' health.

## Introduction

In 1999, *Mental Health: A Report of the Surgeon General* challenged the public health community to define health as a state of complete physical, mental, and social well-being. The report also challenged public health and social service agencies, health care systems, policy makers, communities, and the public to take action to promote mental health for all Americans (1). Integrating mental health and public health programs to prevent chronic disease will require initiating, developing, strengthening, and sustaining public health partnerships with mental health programs at the local, state, and national levels to leverage the strengths and resources of partners and work on common goals (2,3).

*Crossing the Quality Chasm: A New Health System for the 21st Century* (4) and *Improving the Quality of Health Care for Mental and Substance-Use Conditions* (5) describe how the health system could be reinvented to foster innovation, promote prevention and self-care activities, and develop team-based approaches to improve the delivery of care. Similarly, the goal of a transformed public health system is to integrate mental health and physical health so that policies and programs are "person-centered," or more holistic. In this article, we describe examples of partnership projects that appear promising for incorporating mental health promotion into public health promotion. We draw these examples from the national, state, and local or community levels (6,7) (Table). Successful partnerships include participation by representatives from public health programs related to chronic disease prevention and control, mental health and primary care providers, and community members, including mental health advocates. Additional stakeholders include academic institutions, substance abuse counselors, faith-based communities, and business, civic, and political leadership.

## Partnership Projects

On the national level, a federal partnership between the Substance Abuse and Mental Health Services Administration (SAMHSA) and the Centers for Disease Control and Prevention (CDC) is developing and implementing mental health and mental illness modules in the Behavioral Risk Factor Surveillance System (BRFSS). These modules provide data on state-level estimates of mental health (8,9) and assess the associations of mental health and mental illness to chronic diseases and health risk behaviors (10). On the state level, partnerships have been established among multiple offices and divisions within the Maine Department of Health and Human Services, an academic center, and stakeholder groups to integrate health issues into a mental health system designed to improve diabetes health outcomes for people with serious mental illness.

On the community level, a Prevention Research Center has partnered with local aging and disability agencies and other community organizations in Seattle/King County, Washington, to modify an existing home health service to reduce depression and improve the quality of life among socially isolated, chronically ill seniors (6). The second community-level example is a partnership between the New York City Department of Health and Mental Hygiene, municipal hospitals, and the New York Business Group on Health to promote depression screening and management as standard practice and to secure coverage for this service in all primary care settings in New York City (7).

The elements necessary to bring about change have been well described and are applicable to an effort to integrate mental health and public health policy and programming (11):

1. Make the case for the need for change through epidemiologic surveillance partnership efforts. Such efforts should coordinate the collection, analysis, and dissemination of data on the interrelationships between mental illness, health risk behaviors, and chronic diseases and their effect on the health of specific populations. For example, those who initiated the Maine project produced and disseminated BRFSS and Medicaid data on the interactions among mental illness, health risk, and chronic disease and their effect on health outcomes as well as use and cost of services. These reports gained support from senior administrators from state Medicaid, mental health, and public health agencies to promote integration of mental and physical health. Critical to the success of this effort were state-level data that showed partners how addressing mental health can advance their core mission and objectives.
2. Recruit a "champion" at every level. Change requires motivation from the top, but at least 1 champion is needed at each level to initiate, implement, and sustain change. The federal agency partnership resulted from CDC's interest in expanding BRFSS to encompass mental health and SAMHSA's initiative to improve monitoring of the mental health status of the nation. Operational staff members in SAMHSA and CDC collaborated and implemented the project with the full support of both agencies. Both agencies also recruited change agents at the state level.
3. Form a team by identifying and assembling the relevant change agents into a working group. The original champion needs to recruit other champions and partner groups; the effort cannot be sustained if it is seen as the special work of 1 or 2 people. Collaborations involving multiple agencies and organizations enable leveraging of resources that increase the likelihood of sustainability. The Take Care New York Depression Initiative brought together multiple partners to promote depression screening and management in primary care settings as well as to implement a public relations campaign, "Have You Asked Your Doctor About a Test for Depression?" The municipal hospitals, voluntary hospitals, and community health centers were recruited to embed depression screening in their existing primary care clinics and electronic medical records. A common screening instrument was proposed by the Department of Health and Mental Hygiene. The New York City Public Health Agency and its Department of Aging used their existing resources to provide public health nursing outreach, educational workshops, screening, referral, and follow-up for public health clinic clients and the elderly. The New York Business Group on Health was recruited to encourage member organizations and insurance providers to require standardized depression screening in primary care to maintain, expand, and sustain the integration of mental health into health practice.
4. Develop local projects with time-specific, measurable objectives that are related to a specific population and select where positive outcomes and early wins can be achieved. All examples in the Table had interventions and activities that were simple, targeted, and within the scope of the missions, resources, infrastructures, processes, and existing programs of the partners.
5. Track and evaluate changes by establishing measures and collecting the appropriate, and often new, data to allow monitoring and evaluation of the effect of this partnership on systems and health outcomes. Evaluation may also include assessing improvements in access to and satisfaction with care or services, determining the effect of policy changes on outcomes or services, and making the case for cost-effectiveness (the return on investment in this effort). In partnership with Seattle's Aging and Disability Services and Senior Services of Seattle/King County, the Program to Encourage Active, Rewarding Lives for Seniors (PEARLS), a randomized control trial in Seattle/King County, aimed to reduce minor depression and resulting disability among older adults by teaching them depression-management techniques. The project was evaluated in terms of depression and quality of life 12 months later (6). Compared with the usual care group, patients who received the PEARLS intervention were significantly more likely to have a 50% or more reduction in depressive symptoms, achieve complete remission from depression, and have more functional and emotional well-being. No difference was seen, however, in health care use such as outpatient visits, emergency department visits, or hospitalizations. Total mean cost per patient for PEARLS during the 12 months was $630.

## Sustainability

The partnerships described here have emerged only in the last 5 years and are as yet fledging efforts. The next phase requires development of strategies for maintaining and expanding these promising efforts. Multiple studies point to factors that can improve sustainability (12-15). Steps that advance sustainability include participatory engagement with stakeholders, where all stakeholders contribute collaboratively to a strategic planning process. Partnerships that maintain healthy relationships — high levels of trust, reciprocity, and respect — have better chances of being sustainable (12,13).

Also needed for sustainability is institutional support, where the integration of mental health and health issues is embedded throughout the organization, for example, in strategic planning, messaging, standards, accountability, organizational charts, job descriptions, and contract language. Projects aligned with existing institutional priorities or leading to institutional policy changes are more likely to be sustained.

Process and outcomes measures that can demonstrate positive benefits relevant to each of the partners increase the likelihood that the programs will be sustained (13). Projects are also more likely to be replicated if there is broad dissemination of outcome data tailored to the interests of each of the stakeholders. For partnerships promoting integration, evaluation activities should address personal-level outcomes across both mental and physical health, with the ultimate goal of demonstrating shared benefits.

Programs that have a "train-the-trainer" component are more sustainable than are those without training components. Trained staff members continue to provide program benefits and consistency (12). The development of sustainable training programs should target all partners, including workforce, consumers, and community leaders, and should address both health and mental health competencies.

Partnerships that invest resources for maintenance and recurring costs are more likely to be sustained (12-14). This investment may involve reallocation of funds or personnel, pooled funding from multiple partners, or giving permission in existing public or mental health funding streams or block grants to create mental health or health deliverables. It may also involve support for parity legislation, advocacy with employers who pay for health insurance, and adopting rules regarding changes in reimbursement to support integration of mental health in primary care sites.

## Conclusions

Integration of mental health and chronic disease public health programs is a challenging but essential task in protecting Americans' health. The examples in this article illustrate the role of partnership in achieving this goal. Especially in times of limited resources, partnerships can capitalize on existing programs and develop new ideas that make the most of smaller budgets. Synergistic integration of activities for mental and public health will be more effective than individual stakeholder efforts.

## Acknowledgments

We appreciate the historical information contributed about partnership examples by the following health professionals: Olinda Gonzalez, Suzianne Garner, Lina Balluz, Ruth Jiles, Tara Strine, Ali Mokdad, Lloyd I. Sederer, Janice Chisholm, Jorge Petit, and Ron Manderscheid.

## Figures and Tables

**Table.** Examples of Partnerships Between Public Health and Mental Health Agencies at the National, State, and Local Levels

**Example and Objectives**	**Partners**	**Major Activities**
**National**		
Design, support, and implement the Anxiety and Depression Module (2006, 2008), which includes the Patient Health Questionnaire 8 and the Mental Illness and Stigma Module (2007, 2009), which includes the Kessler-6 for the state-based Behavioral Risk Factor Surveillance System Support mental health surveillance within a national health survey to obtain mental health surveillance data at the state and local level. Assess the association of mental health and mental illness indicators with health behaviors and chronic diseases. Facilitate and support partnerships at state level between mental health agencies and public health departments.	Funded by the Substance Abuse and Mental Health Services Administration through an interagency agreement with the Centers for Disease Control and Prevention (CDC), which initiated and implemented the project.	Annual and alternate-year implementation of modules. Data analyses, peer-reviewed publications and detailed reports, and data dissemination developed in the states. Technical assistance by CDC to state mental health agencies. Participation of both federal agencies in conferences to integrate mental health and public health.
**State**		
Integration of health issues into mental health system design Improve diabetes health outcomes for people with serious mental illness.	Initiated by the US Department of Health and Human Services Office of Adult Mental Health and Office of Quality Improvement. Partners included the University of Southern Maine Muskie School for support in implementation, reporting, and learning collaboratives as well as senior administrators from state authorities that govern mental health, Medicaid, public health and facility licensing to ensure integration of project successes into policy, regulation, reimbursement, and contracting. A stakeholder group included representation from mental health and primary care providers, community public health partners, and consumer advocacy groups to oversee implementation activities.	Integrate Medicaid care management with mental health case management. Develop systems for tracking health risk and care outcomes in the mental health systems. Educate consumers and mental health workforce in health literacy, disease self-management, and health and wellness. Support communication between primary care and mental health centers. Train consumers in becoming peer partners for other consumers. Leverage resources in local public health activities.
**Local**		
Program to Encourage Active, Rewarding Lives for Seniors (PEARLS) Promote a home-based intervention involving problem-solving treatment to reduce depression among socially isolated, chronically ill seniors. Examine improvements in depression and quality of life as well as changes in health care use.	Initiated and developed by the University of Washington in partnership with social workers and therapists at Aging and Disability Services, Senior Services of Seattle/King County, and other community organizations that focused on the elderly.	Social workers administered screening tool to >370 potential participants to identify eligible clients with depression, 150 patients were eligible, and 138 agreed to enroll in the study. Three home health therapists were trained in and implemented problem-solving techniques with home-bound seniors to increase patients' interactions outside the home and encourage group activities. Changes in depression among participants were tracked by using the Patient Health Questionnaire 9.
Take Care New York Depression Initiative: Get Help for Depression Promote depression screening and management as standard practice in all primary care settings in New York City. Recommend use of the Patient Health Questionnaire 9. Increase treatment for depression among New Yorkers by 10% by 2008.	Initiated in 2004 and launched in October 2007 by New York City Department of Health and Mental Hygiene. Partners included New York City's municipal hospitals (Health and Hospitals Corporation) to embed depression screening into primary care clinics and electronic medical records; the New York City Department for Aging in providing workshops, screening, referral, and follow-up for the elderly; and the New York Business Group on Health to encourage member organizations and insurance providers to reimburse or support standardized depression screening in primary care.	Visit all primary care physicians in highest-risk communities to make recommendations and instruct them on depression screening and supply clinical management tools, such as guidelines and patient self-care techniques. Implement a public relations campaign, "Have You Asked Your Doctor About a Test for Depression?"
